# Prevalence and Diversity of *Bartonella* Species in Commensal Rodents and Ectoparasites from Nigeria, West Africa

**DOI:** 10.1371/journal.pntd.0002246

**Published:** 2013-05-30

**Authors:** Joshua Kamani, Danny Morick, Kosta Y. Mumcuoglu, Shimon Harrus

**Affiliations:** 1 Parasitology Division, National Veterinary Research Institute Vom, Plateau State, Nigeria; 2 Koret School of Veterinary Medicine, The Hebrew University of Jerusalem, Rehovot, Israel; 3 Department of Microbiology and Molecular Genetics, The Kuvin Center for the Study of Infectious and Tropical Diseases, Hebrew University Hadassah Medical School, Jerusalem, Israel; University of Texas Medical Branch, United States of America

## Abstract

**Background:**

Bartonellae are fastidious bacteria causing persistent bacteremia in humans and a wide variety of animals. In recent years there is an increasing interest in mammalian bartonelloses in general and in rodent bartonelloses in particular. To date, no studies investigating the presence of *Bartonella* spp. in rodents and ectoparasites from Nigeria were carried out.

**Methodology/Principal Findings:**

The aim of the current study was to investigate the presence of *Bartonella* spp. in commensal rodents and their ectoparasites in Nigeria. We report, for the first time, the molecular detection of *Bartonella* in 26% (46/177) of commensal rodents (*Rattus rattus, R. norvegicus* and *Cricetomys gambianus*) and 28% (9/32) of ectoparasite pools (*Xenopsylla cheopis*, *Haemolaelaps* spp., *Ctenophthalmus* spp., *Hemimerus talpoides*, and *Rhipicephalus sanguineus*) from Nigeria. Sequence analysis of the citrate synthase gene (*gltA*) revealed diversity of *Bartonella* spp. and genotypes in Nigerian rodents and their ectoparasites. *Bartonella* spp. identical or closely related to *Bartonella elizabethae, Bartonella tribocorum* and *Bartonella grahamii* were detected.

**Conclusions/Significance:**

High prevalence of infection with *Bartonella* spp. was detected in commensal rodents and ectoparasites from Nigeria. The *Bartonella* spp. identified were previously associated with human diseases highlighting their importance to public health. Further studies need to be conducted to determine whether the identified *Bartonella* species could be responsible for human cases of febrile illness in Nigeria.

## Author Summary

*Bartonella* species are zoonotic vector-borne bacteria that typically parasitize the erythrocytes of mammalian hosts, resulting in long lasting infections. They are responsible for a wide range of clinical manifestations in both immunocompetent and immunocompromised hosts.

Rodents and a wide range of small mammals serve as reservoirs of bartonellae, usually with no apparent clinical manifestations. Close association between rodents and humans especially in rural communities as well as in the overcrowded cities facilitates transmission of these bacteria.

There have been no studies investigating the presence of *Bartonella* spp. in rodents and ectoparasites from Nigeria. The aim of the current study was to investigate the presence of *Bartonella* spp. in commensal rodents and their ectoparasites in Nigeria and its public health implications. We report, for the first time, the molecular detection of *Bartonella* in 26% (46/177) of commensal rodents and 28% (9/32) of ectoparasite pools from Nigeria. Sequence analysis of the citrate synthase gene (*gltA*) revealed diversity of *Bartonella* spp. and genotypes in Nigerian commensal rodents and their ectoparasites. The *Bartonella* spp. detected in this study were identical or closely related to *Bartonella elizabethae*, *Bartonella tribocorum* and *Bartonella grahamii* previously associated with human diseases highlighting their importance to public health.

## Introduction

Bartonellae are Gram-negative facultative intracellular alpha-proteobacteria belonging to the family Bartonellaceae. Many *Bartonella* species have been affecting human life for centuries [1]. Since the first *Bartonella* species discovery, namely *Bartonella bacilliformis*, in 1905 by Alberto Leonardo Barton Thompson, more than 30 species of *Bartonella* were identified [2], [3]. *Bartonella* species have been found in a variety of mammals, and the numbers of *Bartonella* species and their respective reservoir hosts are constantly growing [4]. They are pathogens of emerging and reemerging significance, causing a wide array of clinical syndromes in human and animal hosts [5].

These bacterial species are transmitted between the reservoir and the final mammal host by hematophagous arthropods and insects such as fleas, sand flies, mites, lice and possibly ticks, usually by their bites [6]–[8]. The range of vectors involved in the transmission of the different species of this genus has not been fully characterized [9]. Bacteria belonging to the genus *Bartonella* are slow growers *in vitro*, and the most used diagnostic methods are isolation, serology and polymerase chain reaction (PCR). The use of sequencing on PCR amplicons has been recommended in order to detect new species, especially when dealing with uncommon clinical presentations and settings [4].

*Bartonella* DNA has been detected in various hosts and possible vectors in many countries including, Israel [10], [11], Indonesia [12], Nepal [13], Thailand [6], [14], China [15], Taiwan [16], Korea [17], USA [18]–[20], UK [21], [22], Spain [23] and The Netherlands [24]. In Africa there are reports from Kenya [25], the Democratic Republic of Congo and Tanzania [26], Algeria [27], [28], Egypt [29], [30], Gabon [31] and South Africa [32], [33]. However, there is no report of molecular screening of humans or animals and their ectoparasites for *Bartonella* spp. in Nigeria.

Although there are no case estimates of fever of unknown origin (FUO) in Nigeria, the condition remains a challenging medical problem and unraveling the diagnosis could be a daunting task when investigating for common infective and non-infective causes. Moreover, since *Bartonella* spp. are difficult to diagnose and are seldom included in the differential diagnosis list in cases of FUO, specific *Bartonella* sp. treatment is rarely instituted to patients with FUO.

The objectives of this study were to determine the possible infection of commensal rodents and their ectoparasites from Nigeria with *Bartonella* spp., to investigate the presence of zoonotic *Bartonella* spp. in these rodents and ectoparasites and to evaluate genetic heterogeneity of circulating *Bartonella* strains in this country.

## Materials and Methods

### Ethics statement

The study protocol was read and approved by The National Veterinary Research Institute Vom Ethical Committee on Animal Use and Care. Permission to place the traps in the study area was granted by the residents. Animals were treated in a humane manner and in accordance with authorizations and guidelines for Ethical Conduct in the Care and Use of Nonhuman Animals in Research of the American Psychological Association (APA) for use by scientists working with nonhuman animals (American Psychological Association Committee on Animal Research and Ethics) in 2010.

### Rodents and ectoparasites

Rodents were live trapped in domestic and peri- domestic areas in Vom (9°44′N/8°47′E) Nigeria between October–December 2011. A total of 177 rodents (48 *Rattus rattus*, 121 *Rattus norvegicus*, 6 *Mus musculus* and 2 *Cricetomys gambianus*) were captured. Trapping was done using wire cage traps baited with smoked fish and other food scraps set out in the evenings when rodents are known to leave their holes to scavenge in farmlands or nearby human habitations. Traps were checked for rodents early the next morning. Cages containing rodents were transported to the Parasitology Laboratory, National Veterinary Research Institute (NVRI) Vom Nigeria, where they were identified and classified by a zoologist. At the laboratory, the cages containing rodents were placed into a clear plastic bag, which was sealed at the opening. Halothane gas was applied into the bag and the activity of the rodents was monitored. Once the rodents were anaesthetized, they were removed from the cage and bled by cardiac puncture. Depending on the size, 0.5–3 ml of blood was drawn and aliquoted into an EDTA tube and labeled. Each rodent was checked for ectoparasites by brushing the fur with a tooth brush onto a white cardboard paper. Ectoparasites were placed in labeled vials containing absolute ethanol corresponding to the host from which they were removed and stored at −20°C. Both blood and ectoparasite samples were transported in a cool box to The Koret School of Veterinary Medicine, The Hebrew University of Jerusalem, Israel for analysis. The ectoparasites were morphologically identified by an entomologist (KYM) at the Department of Microbiology and Molecular Genetics in Jerusalem, Israel.

### *Bartonella* culture

Two hundred microlitres of thawed whole blood sample was plated onto chocolate agar. The plate was incubated at 35°C and 5% CO_2_ and checked for growth of *Bartonella* species on alternate days for up to 30 days. Suspected colonies were randomly selected and separately sub-cultured onto different fresh agar plates to obtain pure colonies.

### DNA extraction from rodent blood

DNA was extracted from blood using Bi*O*stic Bacteremia DNA Isolation Kit (MO Bio Laboratories, Inc USA) according to manufacturer's instructions.

### DNA extraction from ectoparasites

The ectoparasites collected from each rodent species were pooled (2–3 arthropods per pool) according to genus and/or species. DNA was extracted from each pool using Illustra tissue and cell genomic Prep miniSpin kit (GE Healthcare UK Limited) according to manufacturer's instructions.

### DNA extraction from bacterial cultures

Pure cultured colonies of *Bartonella* sp. were aseptically scooped into microfuge tubes containing 50 µl of sterile Phosphate Buffered Saline (PBS). DNA was extracted from the bacterial colonies using Illustra tissue and cell genomic Prep miniSpin kit (GE Healthcare UK Limited) according to the manufacturer's instructions.

### PCR assays for *Bartonella* sp. citrate synthase gene (*gltA*) from blood and ectoparasites

The oligonucleotide primers: forward BhCS871.p (5′ -GGGGACCAGCTCATGGTGG-3′) and reverse: BhCS1137.n (5′-AATGCAAAAAGAACAGTAAACA-3′) [34] were used for amplification of a 379 bp region of the *Bartonella* citrate synthase gene (*gltA*). Positive and negative controls were included in each PCR run. PCR was performed using reaction tubes, preloaded with a premier PCR master mix (Syntezza PCR-Ready High Specificity, Syntezza Bioscience, Israel). 50 µl total volume was used as follows: 3 µl of DNA template, 1 µl of 10 mM each primer, 1 µl MgCl_2_, 19 µl of ultra pure PCR water and 25 µl PCR master mix. Amplification was performed using a conventional thermocycler (Biometra, Goettingen, Germany) and the following program parameters: an initial denaturing at 95°C for five minutes, and 35 cycles of denaturation at 95°C for one minute, annealing at 56°C for one minute, and elongation at 72°C for one minute. Amplification was completed by holding the reaction mixture at 72°C for 10 minutes.

PCR products were tested for the presence of amplicons of the correct size by electrophoresis of 6 µl of the products on 1.5% agarose gels stained with ethidium bromide and checked under UV light for the size of amplified fragments by comparison to a 50 bp DNA molecular weight marker. Amplicons of the proper size were identified by comparison to the positive control lane on the gel.

### Sequencing and analysis of DNA

Positive PCR products were purified using (EXO-SAP IT USB, Cleveland, Ohio, USA) and **s**equenced using the forward primer at the Center for Genomics Technologies, Hebrew University of Jerusalem, Israel. To avoid errors or misinterpretation of the sequencing results, we deleted primer sequences from the *gltA* sequences and removed all ambiguities in the sequences before sequence analysis was performed.

### Phylogenetic analysis

Analysis of DNA sequences and phylogenetic relationships were done using MEGA 5.

Sequences were aligned by MUSCLE and the evolutionary history was inferred using the Maximum Likelihood method based on the Tamura-Nei model [35]. The bootstrap consensus tree inferred from 200 replicates was taken to represent the evolutionary history of the taxa analyzed. Branches corresponding to partitions reproduced in less than 50% bootstrap replicates are collapsed. The percentage of replicate trees in which the associated taxa clustered together in the bootstrap test are shown next to the branches. All positions containing gaps and missing data were eliminated.

## Results

### Blood

A total of 177 cardiac blood samples from four rodent species were examined in this study: 68.4% (121/177) from *Rattus norvegicus* rats; 26% (48/177) from *Rattus rattus* rats; 1.1% (2/177) from *Cricetomys gambianus* rats, and 3.4% (6/177) from *Mus musculus* mice.

### Ectoparasites

One hundred and seventy ectoparasites comprising of 85 ticks, *Rhipicephalus sanguineus* (79) and *Haemaphysalis leachi* (6); 13 fleas, *Xenopsylla cheopis* (8), *Ctenophthalmus* spp. (5) and 62 *Haemolaelaps* spp. (gamasid mites) were recovered from the rodents. Ten additional *Hemimerus talpoides* (earwig sp.) were removed from the 2 *C. gambianus* captured (Table 1).

**Table 1:** Number and percentage of *Bartonella* positive ectoparasites as determined by PCR targeting *gltA* fragment.

S/no	Ectoparasite type	Number of pools tested	Number positive	Percent
1	*Xenopsylla cheopis*	2	1	50.0
2	*Ctenophthalmus* sp.	3	2	66.7
3	*Haemolaelaps* spp.	14	3	21.4
4	*Rhipicephalus sanguineus*	10	1	10.0
5	*Hemimerus talpoides*	2	2	100.0
6	*Haemaphysalis leachi*	1	0	0
**Total**		**32**	**9**	**28.1**

### *Bartonella* sp. culture

Due to contamination problems, bartonellae could be cultured from a small subset of 30 rodent blood samples only. Nine of the latter 30 blood samples produced typical bartonellae growth. The colonies were creamy white in color, small, moist with metallic sheen and tended to pit on the agar. Initial growth of *Bartonella* sp. cultures were seen after 5–7 days of incubation. Colonies were sub cultured onto new plates to obtain pure cultures, which were harvested and preserved in 10% glycerol at −80°C until molecularly analyzed.

### Detection of citrate synthase gene (*gltA*) fragments in rodent blood and ectoparasites

*Bartonella gltA* gene fragments were detected in 46 of 177 (26%) rodent blood samples screened in this study. One of 2 *C. gambianus* (50%), 36 of 121 *R. norvegicus* (29.8%), and 9 of 48 *R. rattus* (18.8%) were positive for *Bartonella* sp. DNA. None of the 6 *M. musculus* examined was positive for *Bartonella* sp. *gltA*. Nine of 32 (28%) ectoparasite pools removed from 48/177 (27.1%) rodents were positive for *Bartonella gltA* DNA. All the ectoparasite species tested were positive for *Bartonella* sp. *gltA* except *H. leachi* (Table 1).

Forty six *gltA* sequences were obtained from blood, 3 from bacterial cultures and 9 from ectoparasite samples. Selected *Bartonella* sequences were deposited in GenBank under the following accession numbers: JX0265667–JX0265697 for blood, JX026972 for culture, and JX 026997–JX027006 for ectoparasites.

### Comparison of retrieved *Bartonella gltA* sequences from rodent blood with GenBank deposits

Sequences obtained were compared with *Bartonella* sp. sequences deposited in GenBank for sequence similarity. Thirty six sequences were obtained from *R. norvegicus* blood, 26 of which had 98–100% identity with GenBank deposited *B. elizabethae* sequence (n = 2,100% identity; n = 23, 99%; n = 1, 98%). Nine of the sequences obtained from *R. norvegicus* blood had 97–98% identity with GenBank deposited *Bartonella tribocorum* sequence, while 1 sequence had 98% similarity with GenBank deposited *Bartonella grahamii*. Nine sequences were obtained from *R. rattus* blood, 7 of which had sequence identity of 98–100% with GenBank deposited *B. elizabethae* sequence (n = 3, 100% identity; n = 1, 99%; n = 3, 98%) (Table 2). The sequence retrieved from the blood of *C. gambianus* had 99% identity with GenBank deposited *B. elizabethae*.

**Table 2:** Genetic relationship between *Bartonella* species detected in this study and those from other geographic regions.

Genotypes determined in this study with deposited accession number	Host in this study	First match identity with GenBank deposited sequence accession number. and similarity percentage	Host of related sequence	Country of host	Attributed to
RN 149-JX026969	*Rattus norvegicus*	*Bartonella* sp. (EF213769.1); 99	*Rattus norvegicus*	China	*Bartonella elizabethae*
RN4-JX026970	*Rattus norvegicus*	Uncultured *Bartonella* sp. (FJ686050.1);99	*Acomys cahirinus*	Israel	*Bartonella elizabethae*
RR28-JX026971	*Rattus rattus*	Uncultured *Bartonella* sp. (FJ851115.1);99	Small mammals	Equatorial Africa	*Bartonella elizabethae*
RN 128-JX026972	*Rattus norvegicus*	*Bartonella elizabethae* (GQ225710.1);99	Humans	Thailand	*Bartonella elizabethae*
CG169-JX026973	*Cricetomyces gambianus*	Uncultured *Bartonella* sp. (FJ686050.1);99	Wild rodents	Israel	*Bartonella elizabethae*
RN171-JX026976	*Rattus norvegicus*	*Bartonella elizabethae* (GQ225710.1);98	Humans	Thailand	*Bartonella elizabethae*
RN 132-JX026981	*Rattus norvegicus*	*Bartonella tribocorum* (AM260525.1);97	NA	NA	*Bartonella tribocorum*
RN 10-JX026990	*Rattus norvegicus*	Uncultured *Bartonella* sp. (FJ851112.1);98	Small mammal	Equatorial Africa	*Bartonella grahamii*
RN143-JX026977	*Rattus norvegicus*	Uncultured *Bartonella* sp. (FJ686050.1);99	Wild rodent	Israel	*Bartonella elizabethae*
RR159-JX026974	*Rattus rattus*	*Bartonella* sp. FJ589062.1);99	*Rattus tanezumi flavipectus*	China	*Bartonella elizabethae*
RN5-JX026978	*Rattus norvegicus*	Uncultured *Bartonella* sp.(FJ851112.1);97	Small mammal	Equatorial Africa	*Bartonella elizabethae*
RR164-JX026968	*Rattus rattus*	*Bartonella* sp. (FJ589062.1);100	*Rattus tanezumi flavipectus*	China	*Bartonella elizabethae*
RT20-JX026997	*Xenopsylla cheopis*	*Bartonella* sp. (FJ589056.1);98	*Rattus tanezumi flavipectus*	China	*Bartonella elizabethae*
RT22-JX027008	*Ctenophthalmus sp.*	Uncultured *Bartonella* sp. (FJ851112.1); 97	Small mammal	Equatorial Africa	*Bartonella tribocorum*
RT7-JX027005	*Rhipicephalus sanguineus*	*Bartonella elizabethae* (GQ225710.1); 97	Human	Thailand	*Bartonella elizabethae*
RT30-JX026999	*Hemimerus talpoides*	Uncultured *Bartonella* sp.(FJ686050.1); 99	*Acomys cahirinus*	Israel	*Bartonella elizabethae*

### Comparison of retrieved *Bartonella gltA* sequences from ectoparasites with GenBank deposits

*Bartonella gltA* sequences obtained from one pool each of *X. cheopis*, *R. sanguineus*, and 3 pools of *Haemolaelaps* sp. had 97–100% similarity to *B. elizabethae* deposited in GenBank while a sequence from *Ctenophthalmus* sp. pool had 97% identity with *B. tribocorum* sequence deposited in GenBank. Interestingly, *Bartonella* sp. DNA with 99% sequence identity to *B. elizabethae* deposited in the GenBank was detected from one pool of *H. talpoides* earwigs that were removed from *C. gambianus* rats.

### Comparison of *Bartonella* DNA in ectoparasites and their hosting rodents

*Bartonella* spp. DNA was detected in 4 of 13 (30.8%) rodents from which the ectoparasites were removed. However, only one ectoparasite, *H. talpoides* removed from *C. gambianus* had the same percent sequence identity (100%) with that of the host. The DNA sequences from the ectoparasites had 97–99% identity with their first GenBank match (Table 3). The *R. sanguineus* pool that was positive for *Bartonella* spp. DNA was collected from *R. norvegicus* rat that was negative for *Bartonella* sp. DNA.

**Table 3:** Sequence similarity between *Bartonella* sp. DNA from ectoparasites and their hosting rodent.

S/No	Ectoparasite type	GenBank first match, accession number and percentage similarity	
		Ectoparasite	Host
1	*Mesostigmata*	Uncultured *Bartonella* sp. (FJ686050.1); 99	Negative
2	*Mesostigmata*	Uncultured *Bartonella* sp.(FJ851115.1); 99	*Bartonella* sp. (EF213769.1); 99
3	*Xenopsylla cheopis*	*Bartonella* sp. (FJ589056.1)98%	Uncultured *Bartonella* sp. (FJ686050.1); 99
4	*Rhipicephalus sanguineus*	*Bartonella elizabethae* (GQ225710.1) ; 97	Negative
5	*Ctenophthalmus* sp.	Uncultured *Bartonella* sp. (FJ851112.1); 97	Uncultured *Bartonella* sp. (FJ686050.1); 99
**6**	*Hemimerus talpoides*	Uncultured *Bartonella* sp. (FJ686050.1); 99	Uncultured *Bartonella* sp. (FJ686050.1); 99

### Phylogenetic analysis of *gltA* sequences

**Figure 1:**
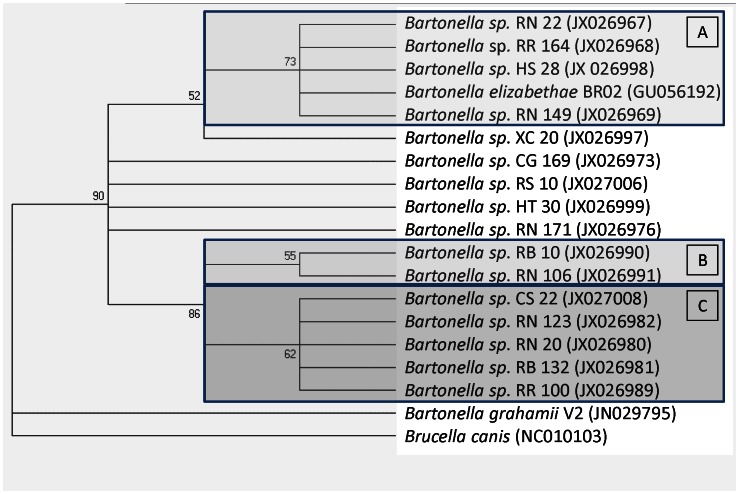
Phylogenic tree of *Bartonella gltA* sequences detected in this study showing three distinct clusters A–C.

The phylogenetic relationship among the genotypes obtained in the present study and previously described *Bartonella* species is presented in Figure 1. Sequences of *Bartonella* sp. from this study formed 3 distinct clusters A-C along with *B. elizabethae* and *B. grahamii* (Fig. 1), but was distantly related to other sequences deposited in the GenBank. The first cluster (cluster A) consists of 4 sequences closely related to *B. elizabethae*. However, 5 other sequences that were 97–100% similar to *B. elizabethae* appear as single genotypes just below cluster A. Cluster B is made up of 2 sequences that were similar to *B. grahamii* deposited in the GenBank. The cluster C consists of 5 sequences that were 97–100% similar to *B. tribocorum* deposited in the genBank.

Sequences were coded based on rodent or ectoparasites species from which they were detected, accession numbers are in parentheses; RR = *Rattus rattus*; RN = *Rattus norvegicus*; CG = *Cricetomys gambianus*; CS = *Ctenophthalmus* sp; HT = *Hemimerus talpoides*; RS = *Rhipicephalus sanguineus*, MS = *Haemolaelaps* spp.; XC = *Xenopsylla cheopis*.

## Discussion

In this study, we report the molecular detection and genetic characterization of *Bartonella* species in rodents and ectoparasites from Nigeria, West Africa. Moreover, to the best of our knowledge, this is the first report of molecular investigation of *Bartonella* spp. in rodents and their ectoparasites in this country. The 26% prevalence of *Bartonella* DNA found in this study was higher than the 8.5% prevalence reported in small mammals from the Democratic Republic of Congo but lower than and 38% reported in Tanzania [26]. The differences between the findings in the latter studies and ours can be attributed to the fact that commensal rodents were screened in the current study while sylvatic rodents were screened in the DR Congo and Tanzania studies. Similarly, the 28% prevalence of *Bartonella* DNA by *gltA* PCR in ectoparasites in this study was slightly higher than the 21.5% reported in fleas from Algeria, targeting 3 genes and the inter-genic spacer (ITS) [28]. The high prevalence of detection of *Bartonella* spp. DNA in the ectoparasites attests to their role as vectors of these bacteria.

Several *Bartonella* spp. that were associated with human diseases were identified in this study, including *B. elizabethae, B. grahamii and B. tribocorum*. *Bartonella elizabethae* was found in patients with endocarditis [36]. *Bartonella grahamii* was associated with neuroretinitis or bilateral retinal artery branch occlusions [37]. A *Bartonella* genotype closely related (97%) to *B. tribocorum* was detected in the blood of human patient with fever from Thailand [25]. The finding of these zoonotic *Bartonella* spp. in commensal rodents from Nigeria demonstrates their importance as reservoirs for various zoonotic *Bartonella* species and warrants increased awareness of physicians and health care workers for these pathogens especially in unidentified febrile cases.

In this study, no DNA sequence similar to *B. tribocorum* was obtained from *R. rattus* rats. The detection of *B. tribocorum* only in *R. norvegicus* rats is in agreement with the earlier report of Márquez *et al.* [23] which supports the hypothesis that there is specificity of *Bartonella* spp. for their rodent hosts [15].

Although the role of *R. sanguineus* ticks in transmitting *Bartonella* spp. in nature is not proven [38] it is important to note that we detected *Bartonella* DNA in *R. sanguineus* ticks. Detection of *Bartonella* DNA in ticks was previously reported also by other authors [8], [17], [39]. The *Bartonella* spp. DNA detected from one *R. sanguineus* tick pool had 97 percent identity to *B. elizabethae* sequences deposited in GenBank. It is worthy to note that the host from which the *R. sanguineus* ticks were removed was negative for *Bartonella* spp. DNA. This suggests that the ticks might have acquired the bacteria during previous feeding on an infected host. The ability of the tick to transmit this organism to a susceptible host during the next feeding stage or to its progeny is worth further investigation.

Comparative analyses of the *gltA* sequences obtained from *Bartonella* spp. showed that commensal rodents in Nigeria harbor a diverse assemblage of *Bartonella* strains, some of which represent known *Bartonella* spp. and strains and others may represent distinct novel strains. Although only a portion of the citrate synthase gene (*gltA*) was used for phylogenetic analysis, this gene has been shown to be a reliable tool for distinguishing between closely related *Bartonella* genotypes [40]. By using this partial gene, it was possible to compare the variety of *Bartonella* genotypes isolated from rodents with homologous sequences of *Bartonella* strains found in other mammals, reported from other parts of the world. Finding considerable sequence diversity is typical for different species of *Bartonella*, although more characteristics are needed to describe novel *Bartonella* species [3].

In this study, the *Bartonella* genogroups identified in commensal rodents formed three separate clusters closely related to *B. elizabethae* but distantly related to other known *Bartonella* spp. Although BLAST searches shows some of the sequences had 97–100% similarity to *B. tribocorum* and *B. grahamii* sequences deposited in GenBank (Fig. 1). The findings of *Bartonella* sequences that were genetically distant from known GenBank deposited sequences requires further investigation in characterizing these genotypes and ascertaining whether they are pathogenic to animals and/or humans.

Pools of *H. talpoides* collected from *C. gambianus* in this study contained *Bartonella* DNA. *Hemimerus talpoides* (earwig sp.) are presumed to feed on the epidermis of their host or as a saprophytic on fungus from the skin of the host. The detection of *Bartonella* sp. DNA in this ectoparasite is interesting and requires further investigation [41].

In conclusion, this study has resulted in the identification and genetic characterization of *Bartonella* genotypes in commensal rodents and ectoparasites from Nigeria, West Africa. A high prevalence and diversity of *Bartonella* spp. and strains was detected in commensal rodents and their ectoparasites in this study. Several zoonotic *Bartonella* spp. including *B. elizabethae*, *B. grahamii* and *B. tribocorum* were identified for the first time in Nigeria highlighting their importance for public health in this country.
